# Pharmacodynamic effect of bempedoic acid and statin combinations: predictions from a dose–response model

**DOI:** 10.1093/ehjcvp/pvab064

**Published:** 2021-08-27

**Authors:** Satyawan B Jadhav, Ryan L Crass, Sunny Chapel, Michael Kerschnitzki, William J Sasiela, Maurice G Emery, Benny M Amore, P Hugh R Barrett, Gerald F Watts, Alberico L Catapano

**Affiliations:** Ann Arbor Pharmacometrics Group, 900 Victors Way #328, Ann Arbor, MI 48108, USA; Ann Arbor Pharmacometrics Group, 900 Victors Way #328, Ann Arbor, MI 48108, USA; Ann Arbor Pharmacometrics Group, 900 Victors Way #328, Ann Arbor, MI 48108, USA; Daiichi Sankyo Europe GmbH, Zielstattstraße 48, 81379 Munich, Germany; Esperion Therapeutics, Inc., 3891 Ranchero Dr, Ann Arbor, MI 48108, USA; Esperion Therapeutics, Inc., 3891 Ranchero Dr, Ann Arbor, MI 48108, USA; Esperion Therapeutics, Inc., 3891 Ranchero Dr, Ann Arbor, MI 48108, USA; Faculty of Medicine and Health, University of New England, Armidale, NSW 2351, Australia; School of Medicine, University of Western Australia, Medical Research Foundation Building, Rear 50 Murray Street, Perth, WA 6001, Australia; Department of Pharmacological and Biomolecular Sciences, University of Milan and IRCCS Multimedica, Via Balzaretti 9, 20133 Milan, Italy

**Keywords:** Dose response, Drug interaction, Hypercholesterolaemia, LDL cholesterol, Statin

## Abstract

**Aims:**

Many patients are unable to achieve guideline-recommended LDL cholesterol (LDL-C) targets, despite taking maximally tolerated lipid-lowering therapy. Bempedoic acid, a competitive inhibitor of ATP citrate lyase, significantly lowers LDL-C with or without background statin therapy in diverse populations. Because pharmacodynamic interaction between statins and bempedoic acid is complex, a dose–response model was developed to predict LDL-C pharmacodynamics following administration of statins combined with bempedoic acid.

**Methods and results:**

Bempedoic acid and statin dosing and LDL-C data were pooled from 14 phase 1–3 clinical studies. Dose–response models were developed for bempedoic acid monotherapy and bempedoic acid–statin combinations using previously published statin parameters. Simulations were performed using these models to predict change in LDL-C levels following treatment with bempedoic acid combined with clinically relevant doses of atorvastatin, rosuvastatin, simvastatin, and pravastatin. Dose–response models predicted that combining bempedoic acid with the lowest statin dose of commonly used statins would achieve a similar degree of LDL-C lowering as quadrupling that statin dose; for example, the predicted LDL-C lowering was 54% with atorvastatin 80 mg compared with 54% with atorvastatin 20 mg + bempedoic acid 180 mg, and 42% with simvastatin 40 mg compared with 46% with simvastatin 10 mg + bempedoic acid 180 mg.

**Conclusion:**

These findings suggest bempedoic acid combined with lower statin doses offers similar LDL-C lowering compared with statin monotherapy at higher doses, potentially sparing patients requiring additional lipid-lowering therapies from the adverse events associated with higher statin doses.

## Introduction

LDL cholesterol (LDL-C) plays a key role in the development of atherosclerotic plaques and, subsequently, cardiovascular events. Lowering LDL-C levels reduces the risk of atherosclerotic cardiovascular disease proportionally to the absolute reduction in LDL-C.^1,2^ Statins remain the cornerstone of lipid-lowering therapy.^3,4^ Statins competitively inhibit 3-hydroxy-3-methylglutaryl coenzyme A (HMG-CoA) reductase, the rate-limiting enzyme for *de novo* cholesterol synthesis, resulting in up-regulation of hepatic LDL receptors and a reduction in circulating LDL-C.^5^

Many patients with hypercholesterolaemia remain above guideline-recommended LDL-C thresholds despite treatment with maximally tolerated statin doses with or without the addition of non-statin agents (e.g. ezetimibe) and thus remain at elevated risk for cardiovascular disease.^6^ Adverse effects (primarily muscle symptoms) can limit the maximally tolerated statin dose to low-dose therapy, or may make patients not adhere to their treatment or stop their statin therapy completely. Therefore, there is a high unmet need for additional non-statin therapies to help patients achieve lipid-lowering goals.

**Figure 1:**
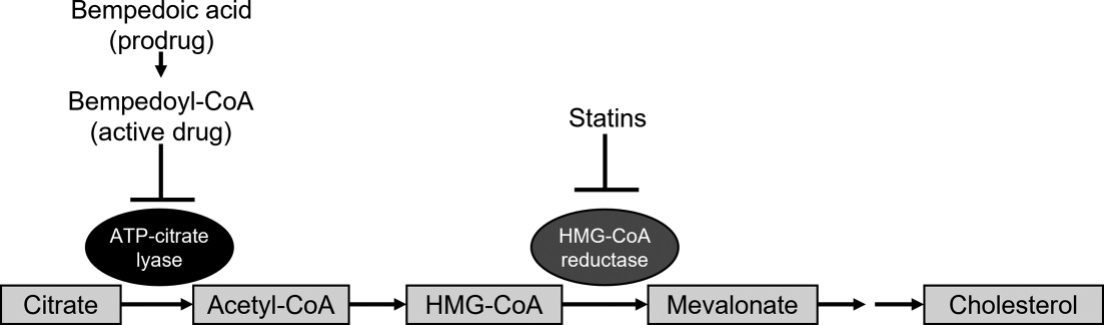
Mechanism of action of bempedoic acid relative to statins. ATP, adenosine triphosphate; HMG-CoA, 3-hydroxy-3-methylglutaryl coenzyme A.

Bempedoic acid, an oral, once-daily medication that lowers LDL-C in patients with hypercholesterolaemia, is approved for use in the United States and Europe with varying indications.^7^ Bempedoic acid is a competitive inhibitor of ATP citrate lyase, an enzyme two steps upstream of HMG-CoA reductase (the target of statins), and lowers LDL-C by decreasing cholesterol synthesis and up-regulating LDL receptors, thus impacting LDL metabolism through this well-established pathway (*Figure 1*). Results from phase 3 trials of bempedoic acid in patients receiving maximally tolerated statins—including patients who were unable to tolerate the lowest approved statin doses due to adverse events—consistently demonstrated clinically meaningful reductions in LDL-C as compared with placebo.^8–12^ Additionally, the degree of LDL-C lowering with bempedoic acid was greater in the pool of statin-intolerant patients receiving low dose, very low dose, or no background statin therapy (82% of whom were not taking statins) compared with the pool of patients who were receiving higher doses of background statins (91% of whom were taking moderate- or high-intensity statins; placebo-corrected least squares mean % change from baseline of −24.5% vs. −17.8%, respectively).^12^ Consistent with the model predictions, in the subset of patients who took no background statin, the placebo-corrected least squares mean LDL-C reduction from baseline was slightly greater (−27.2%) when compared with the higher dose statin or the entire lower dose statin pools.^12^

During phase 3 clinical trials, the addition of bempedoic acid to stable statin therapy was studied; it is important to further evaluate the impact of altering statin doses in combination with bempedoic acid because the pharmacokinetic and pharmacodynamic interaction between statins and bempedoic acid is complex. The objective of this study was to evaluate predictions of LDL-C pharmacodynamics using a dose–response model to assess the potential benefits and risks of adjustments in the statin dose when administered in combination with bempedoic acid.

## Methods

### Data

Data on bempedoic acid dosing and LDL-C concentrations were pooled from 14 clinical studies (1 phase 1,^13^ 4 phase 2,^14–17^ and 3 phase 3 studies^8–10^ with published results and 2 phase 1 and 4 phase 2 studies with data on file) conducted as part of the clinical development of bempedoic acid. Study populations included healthy volunteers (one study) and patients with dyslipidaemia, hypercholesterolaemia, heterozygous familial hypercholesterolaemia, or type 2 diabetes mellitus who received placebo monotherapy, bempedoic acid monotherapy, statin monotherapy, or bempedoic acid in combination with statins. Patients receiving concomitant ezetimibe therapy were excluded from this analysis. Patients receiving stable background therapy with proprotein convertase subtilisin/kexin type 9 (PCSK9) inhibitors with placebo or bempedoic acid from a single study were included in the bempedoic acid monotherapy analysis. A sensitivity analysis was performed to assess the impact of baseline PCSK9 treatment on bempedoic acid monotherapy model parameters.

#### Determination of pre-statin baseline

Patients enrolled into bempedoic acid and statin combination treatment arms were already receiving stable statin therapy at study entry; therefore, their baseline LDL-C concentrations reflect the LDL-C concentration following stable statin therapy. Conversely, baseline LDL-C concentrations in any studies in healthy volunteers or patients receiving bempedoic acid monotherapy represent their baseline prior to any lipid-lowering drugs. To pool these data for analysis, imputations of individual patient baseline LDL-C concentrations measured at study entry were estimated to the ‘pre-statin’ baseline for patients who were receiving stable statin doses prior to combination with bempedoic acid in these trials (see Supplementary material online, *Detailed Methods*).

### Development of dose–response model

#### Overall approach

Indirect-effect dose–response models were developed independently from subject-level data for bempedoic acid monotherapy and for bempedoic acid administered in combination with atorvastatin, rosuvastatin, simvastatin, or pravastatin.

**Figure 2:**
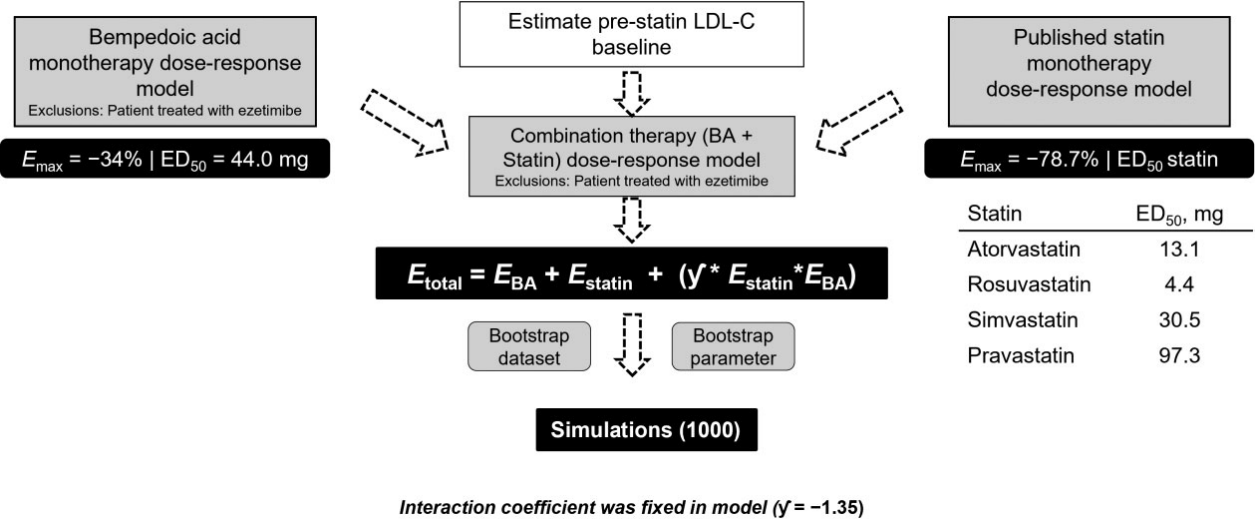
Model-based analysis workflow: bempedoic acid plus statin combination therapy. BA, bempedoic acid; ED_50_, median effective dose; *E*_max_, maximum effective dose; *E*_BA_, efficacy of bempedoic acid; *E*_statin_, efficacy of statin; *E*_total_, total efficacy; LDL-C, LDL cholesterol.

Following identification of the bempedoic acid monotherapy dose–response model, individual bempedoic acid–statin combination dose–response models were developed; model parameters for bempedoic acid response were obtained from the bempedoic acid monotherapy model and those for statin dose response were obtained from a published analysis using trial-level data.^18^ The overall approach is depicted in *Figure 2*. The first‐order conditional estimation with interaction method in NONMEM^®^ (version 7; ICON Development Solutions, Ellicott City, MD, USA) was used for model development. The first-order rate constant describing elimination of LDL-C in the indirect-effect model was fixed to a value consistent with the literature.^19–21^ Post-processing of the results was performed using R (version 3.5, The R Foundation for Statistical Computing, Indianapolis, IN, USA).

### Model evaluation

Model fit of the observed data was assessed visually for each model by overlaying the typical model-predicted LDL-C % change from baseline vs. dose over the corresponding observed mean [95% confidence interval (CI)] values.

### Model-based predictions

Simulations were performed for each of the bempedoic acid–statin combination therapy models to predict LDL-C change from baseline at week 12. Combined bempedoic acid and statin model predictions were further evaluated using posterior predictive checks with parametric bootstrapping of model parameters and non-parametric bootstrapping of the observed datasets used to incorporate uncertainty. The simulated individual predicted change from baseline in LDL-C at week 12 was summarized for statin monotherapy and for bempedoic acid–statin combination therapy for each individual statin and dose combination. The additional LDL-C lowering when bempedoic acid was added to stable background statin therapy at specific doses (ΔLDL-C) was manually computed using the following formula:

$$\begin{eqnarray*}&&[\left( {\% \,\,{\rm{lowering\,\, with\,\, statin}} + {\rm{bempedoic\,\, acid}}} \right)\\
&&\quad-\left( {\% \,\,{\rm{lowering\,\, with\,\, statin\,\, monotherapy}}} \right)]/\\
&&\quad[1\ + (\% \,\,{\rm{lowering\,\, with\,\, statin\,\, monotherapy}}/100)].\end{eqnarray*}$$

Simulations were also performed to predict attainment of absolute LDL-C goals among patients treated with various bempedoic acid–statin combinations. In these simulations, a parametric bootstrap of bempedoic acid–statin combination model parameters was performed. To facilitate comparison across the background statin agents, the population baseline LDL-C and corresponding inter-individual variability parameters from the atorvastatin–bempedoic acid combination model were used for all combinations. This model represents the highest baseline LDL-C condition estimated across background statin therapy. One patient was simulated for each unique dosing condition with each set of parameters resulting in a total of 1000 simulated patients per dose level. Summary statistics of individual predicted LDL-C were tabulated across the 1000 simulated patients in each dosing condition at treatment week 12. Additionally, the proportion of simulated patients achieving clinically relevant LDL-C targets of <100 mg/dL (2.6 mmol/L) and <70 mg/dL (1.8 mmol/L) was determined for each dosing condition and plotted to provide a visual assessment of target attainment.

## Results

### Model parameters

#### Statins

The model parameters for each statin are presented (*Figure 2*). The maximal effect as proportional change from baseline (*E*_max_) and Hill coefficient did not differ among statins, but each statin had a unique dose needed to achieve 50% of maximal effect (ED_50_). All statins shared a similar shape of the dose–response relationship, with a similar maximal effect of 79% reduction in LDL-C over placebo.^18^

#### Bempedoic acid

A simple *E*_max_ model best described the dose–response relationship for bempedoic acid. A sigmoidal *E*_max_ model was tested; however, the Hill coefficient was not precisely estimated with the 95% CI including the null value of 1 at which the sigmoidal *E*_max_ model reduces to a simple *E*_max_ relationship. The maximal reduction in LDL-C with bempedoic acid alone was 34% with an ED_50_ estimated to be 44 mg. All model parameters are shown in *Table 1*. In a sensitivity analysis, exclusion of patients with concomitant PCSK9 inhibitor use did not result in a meaningful difference in model parameters; therefore, patients with concomitant PCSK9 inhibitor use were included in the assessment of bempedoic acid monotherapy (data not shown).

**Table 1:** Bempedoic acid monotherapy dose–response model

Parameter	Estimate	SE	95% CI
Baseline LDL-C, mg/dL	147.1	1.1	(144.9, 149.3)
*k*_out_, h^–1^	0.01	FIX	—
*E*_max-BA_	−0.34	0.01	(−0.36, −0.32)
ED_50-BA_, mg	44.0	3.8	(36.6, 51.3)
Hill_BA_	1 (FIX)	—	—
Residual error			
Proportional, %	8.4	—	(7.5, 9.3)
Additive, mg/dL	11.4	0.6	(10.3, 12.5)
Inter-individual variability, %			
Baseline LDL-C	23.0	—	(21.9, 24.1)
*E*_max-BA_	36.2	—	(32.6, 39.4)

BA, bempedoic acid; CI, confidence interval; ED_50-BA_, dose of bempedoic acid needed to achieve 50% of maximal effect; *E*_max-BA_, maximal bempedoic acid effect as proportional change from baseline; FIX, model parameter fixed to estimated value without error; Hill_BA_, Hill coefficient for bempedoic acid; *k*_out_, first-order rate constant for elimination of LDL cholesterol; LDL-C, LDL cholesterol; SE, standard error.

#### Bempedoic acid–statin interaction

**Figure 3:**
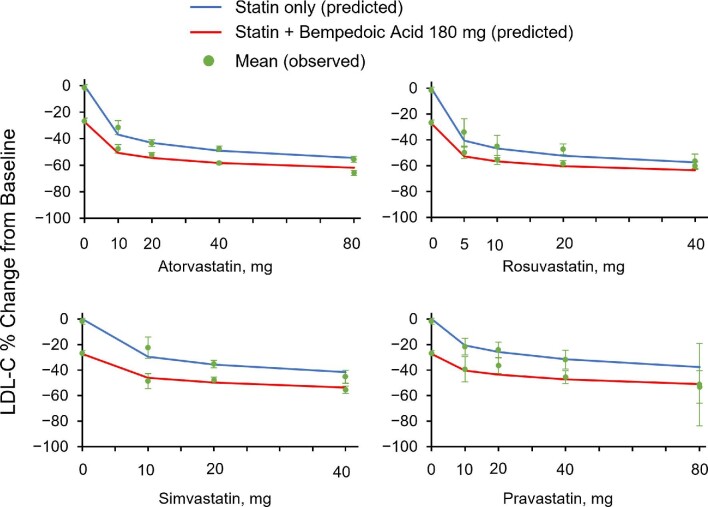
Combination model fit. The observed mean change (red circles) at week 12 is shown with a 95% confidence interval. Solid lines are model-predicted mean change at week 12 for statin monotherapy (blue) and bempedoic acid–statin combinations (red). LDL-C, LDL cholesterol.

Combination models of bempedoic acid with individual statins were developed to predict LDL-C change from baseline using parameters from the bempedoic acid model described earlier and from the published statin dose–response model. The previously published dose–response model parameters for individual statins consisted of a common *E*_max_ (79% reduction from baseline) and unique ED_50_ values for each statin.^18^ Pre-treatment baseline LDL-C values were estimated using these individual statin dose–response model parameters for patients who were treated with maximally tolerated statins before enrolment. Parameters for the combined model with each statin are shown in *Table 2*. Based on sensitivity analysis, the interaction parameter *γ* was fixed to a common value across the individual statins to describe the effect of combination with bempedoic acid. *Figure 3* shows the impact of bempedoic acid 180 mg on the statin dose–response relationship for each statin. In all cases, the model-predicted % LDL-C changes from baseline were within the 95% CI of the corresponding observed mean values, indicating that the models adequately described the observed data. Lowering of LDL-C, in terms of percentage change from baseline, was less than additive with combination therapy. However, the addition of bempedoic acid still provided benefit over individual statin therapy alone.

**Table 2:** Bempedoic acid–statin combination model

Parameter	Atorvastatin	Simvastatin	Rosuvastatin	Pravastatin
Baseline LDL-C, mg/dL	177.9 (176.0, 179.7)	160.4 (158.2, 162.5)	171.9 (169.4, 174.6)	156.7 (154.5, 158.8)
*k*_out_, h^–1^	0.01 (FIX)	0.01 (FIX)	0.01 (FIX)	0.01 (FIX)
*E*_max-statin_	−0.787 (FIX)	−0.787 (FIX)	−0.787 (FIX)	−0.787 (FIX)
ED_50-statin_, mg	13.1 (FIX)	30.5 (FIX)	4.4 (FIX)	97.3 (FIX)
Hill_statin_	0.451 (FIX)	0.451 (FIX)	0.451 (FIX)	0.451 (FIX)
*E*_max-BA_	−0.34 (FIX)	−0.34 (FIX)	−0.34 (FIX)	−0.34 (FIX)
ED_50-BA_, mg	43.96 (FIX)	43.96 (FIX)	43.96 (FIX)	43.96 (FIX)
Hill_BA_	1 (FIX)	1 (FIX)	1 (FIX)	1 (FIX)
*γ*_statin-BA_	−1.35 (FIX)	−1.35 (FIX)	−1.35 (FIX)	−1.35 FIX
Residual error				
Proportional, %	16.1 (15.7, 16.5)	13.5 (12.8, 14.3)	15.1 (14.4, 15.7)	10.0 (8.9, 11.0)
Additive, mg/dL	8.8 (9.5, 8.1)	13.3 (14.3, 12.3)	11.7 (10.6, 12.8)	17.9 (18.9, 16.9)
Inter-individual variability, %				
Baseline LDL-C	27.1 (26.3, 27.8)	25.5 (24.5, 26.5)	30.4 (29.2, 31.4)	24.2 (23.1, 25.2)

Values in parentheses are 95% CI.

Baseline LDL values are from studies of patients with hypercholesterolaemia who were treated with maximally tolerated statins. Patients treated with ezetimibe were excluded from the analysis.

BA, bempedoic acid; ED_50-BA_, dose of bempedoic acid needed to achieve 50% of maximal effect; ED_50-statin_, dose of statin needed to achieve 50% of maximal effect; *E*_max-BA_, maximal bempedoic acid effect as proportional change from baseline; *E*_max-statin_, maximal statin effect as proportional change from baseline; FIX, model parameter fixed to estimated value without error; Hill_BA_, Hill coefficient for bempedoic acid; Hill_statin_, Hill coefficient for statins; *k*_out_, first-order rate constant for elimination of LDL cholesterol; *γ*_statin-BA_, interaction coefficient between statins and bempedoic acid; LDL-C, LDL cholesterol.

### Model-based predictions of LDL-C change from baseline

Model-predicted LDL-C lowering with bempedoic acid 180 mg as monotherapy was −27% (90% CI: −29%, −26%). As shown in *Table 3*, bempedoic acid–statin combination at 25% of the maximum statin dose was predicted to reduce LDL-C levels to a similar or greater extent than maximal dose statin monotherapy (atorvastatin, 54% vs. 54%; simvastatin, 46% vs. 42%; rosuvastatin, 57% vs. 57%; and pravastatin, 44% vs. 38%). At 50% of the maximum statin dose, the bempedoic acid–statin combination provided a reduction in LDL-C levels that was greater than that obtained with maximal dose statin therapy alone (atorvastatin, 58% vs. 54%; simvastatin, 50% vs. 42%; rosuvastatin, 60% vs. 57%; and pravastatin, 47% vs. 38%). ΔLDL-C, which represents the additional LDL-C lowering attributed to bempedoic acid when added to stable background statin therapy at the indicated doses, aligns with data on the LDL-C lowering observed in phase 3 studies where bempedoic acid was added to maximally tolerated background statin therapy.

**Table 3:** Predicted % LDL-C lowering from baseline to week 12 by statins alone and in combination with bempedoic acid

	Mean (90% CI) LDL-C % change from baseline^a^											
	Atorvastatin			Simvastatin			Rosuvastatin			Pravastatin		
Statin dose (mg)	Alone	+ BA 180 mg	ΔLDL-C^b^	Alone	+ BA 180 mg	ΔLDL-C^b^	Alone	+ BA 180 mg	ΔLDL-C^b^	Alone	+ BA 180 mg	ΔLDL-C^b^
0	—	−27 (−29, −26)	—	—	−27 (−29, −26)	—	—	−27 (−29, −26)	—	—	−27 (−29, −26)	—
10	−37 (−40, −30)	−51 (−55, −46)	−22	−30 (−36, −23)	−46 (−50, −42)	−23	−46 (−55, −39)	−57 (−62, −52)	−20	−21 (−26, −16)	−41 (−45, −37)	−25
20	−43 (−51, −36)	−54 (−60, −50)	−19	−36 (−43, −29)	−50 (−54, −45)	−22	−52 (−61, −44)	−60 (−66, −55)	−17	−26 (−32, −20)	−44 (−48, −39)	−24
40	−49 (−57, −41)	−58 (−63, −53)	−18	−42 (−50, −34)	−54 (−59, −49)	−21	−57 (−66, −49)	−63 (−69, −58)	−14	−32 (−38, −25)	−47 (−52, −43)	−22
80	−54 (−63, −46)	−62 (−67, −56)	−17	—	—	—	—	—	—	−38 (−45, −31)	−51 (−56, −46)	−21

BA, bempedoic acid; LDL-C, LDL cholesterol.

a Data represent 1000 simulations using bootstrapped data and parameter estimates.

b ΔLDL-C represents the % reduction in LDL-C due to bempedoic acid when added to stable background therapy at the designated statin dose.

**Figure 4:**
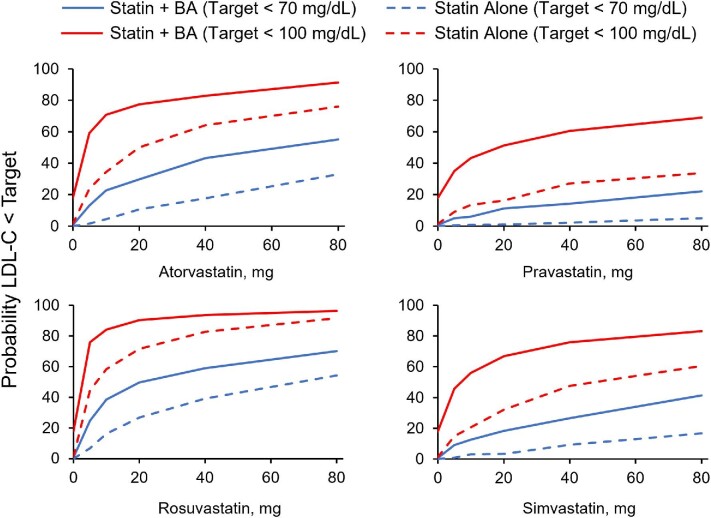
Proportion of patients with LDL cholesterol who achieved LDL cholesterol targets of <100 mg/dL (2.6 mmol/L) and <70 mg/dL (1.8 mmol/L) by statin dose as monotherapy or in combination with bempedoic acid. BA, bempedoic acid; LDL-C, LDL cholesterol.

In terms of predicted absolute LDL-C levels, the model-predicted mean LDL-C baseline with no lipid-lowering therapy was 183.1 mg/dL (4.7 mmol/L). Starting from this common baseline value, the model-predicted mean steady-state LDL-C with bempedoic acid 180 mg alone was estimated to be 132.7 mg/dL (3.4 mmol/L; *Table 4*). With the combination of bempedoic acid and individual statins at 50% of the maximum statin dose, the bempedoic acid–statin combination achieved lower absolute LDL-C levels than did the maximal dose statin administered as monotherapy [e.g. atorvastatin, 77.5 vs. 84.2 mg/dL (2.0 mmol/L vs. 2.2 mmol/L)]. Based on predicted absolute values, the predicted probabilities of achieving LDL-C <100 mg/dL (2.6 mmol/L) and <70 mg/dL (1.8 mmol/L) are depicted in *Figure 4*. The addition of bempedoic acid to statins markedly increased the probability of achieving both LDL-C thresholds.

**Table 4:** Predicted mean absolute LDL cholesterol lowering from baseline to week 12 with bempedoic acid–statin combination

	Mean LDL-C (mg/dL)^a^							
	Atorvastatin		Simvastatin		Rosuvastatin		Pravastatin	
Statin dose (mg)	Alone	+ BA 180 mg	Alone	+ BA 180 mg	Alone	+ BA 180 mg	Alone	+ BA 180 mg
0	183.1	132.7	183.1	132.7	183.1	132.7	183.1	132.7
10	116.2	89.6	129.0	99.6	98.2	79.5	143.4	108.6
20	104.0	84.0	117.7	92.5	87.8	72.6	136.2	104.3
40	94.2	77.5	106.3	86.0	79.0	67.6	124.6	96.9
80	84.2	70.6	—	—	—	—	115.9	90.3

BA, bempedoic acid; LDL-C, LDL cholesterol.

a Data represent 1000 simulation subjects using unique parameter estimates.

## Discussion

Current guidelines recommend the combination of maximally tolerated statins with non-statin agents such as bempedoic acid to intensify LDL-C lowering in patients who cannot achieve desired LDL-C goals with maximally tolerated statin therapy alone.^22–23^ In addition to greater efficacy with combined statin and non-statin therapy compared with either therapy alone, this combination approach may reduce the risk of cardiovascular events while sparing patients from potential side effects of higher statin doses,^22–23^ such as skeletal muscle symptoms and adverse glycaemic effects. This study used a combined bempedoic acid–statin dose–response model to predict LDL-C lowering for bempedoic acid 180 mg combined with various statin doses. Our model showed that adding bempedoic acid to statin therapy is at least equivalent to or more effective in lowering LDL-C than an increase in a statin dose after initial statin treatment. Combinations of bempedoic acid plus the lowest dose of each statin were predicted to achieve similar reductions in LDL-C levels as quadrupling the lowest statin dose as monotherapy. The model predictions suggest that bempedoic acid not only provides additional LDL-C lowering when used in combination with maximally tolerated statins, but also may help maintain the overall LDL-C lowering efficacy when adverse muscle effects need to be managed with statin dose reduction.

Because the data were modelled on a population of patients with a variety of clinical characteristics, the findings would be expected to apply to diverse patient populations. A larger proportion of patients (∼10–40% higher, depending on the statin and statin dose) were predicted to achieve guideline-based LDL-C goals with the combination of statin and bempedoic acid at all levels of statin dosing compared with statin monotherapy, which demonstrates the magnitude of benefit likely to be gained from the addition of bempedoic acid to a statin regimen. Triple combination therapy with bempedoic acid, a statin, and ezetimibe may provide further LDL-C lowering. A recent study showed mean LDL-C lowering from baseline with bempedoic acid 180 mg + ezetimibe 10 mg + atorvastatin 20 mg after 6 weeks of treatment was −63.6%.^24^ In comparison, LDL-C reductions from baseline at week 12 were −36.2% with bempedoic acid 180 mg + ezetimibe 10 mg^25^ and in our model −54% (90% CI: −60%, −50%) with bempedoic acid 180 mg + atorvastatin 20 mg.

Model predictions of LDL-C lowering achieved with combination therapy appeared to be less than additive based on the value of the interaction coefficient (*γ*) identified in a sensitivity analysis. This finding is consistent with the site of action of bempedoic acid, which is upstream of HMG-CoA reductase in the cholesterol biosynthetic pathway and may limit the potential for completely additive pharmacodynamic effects of the combination. The narrowing difference in lowering LDL-C with increasing statin dose between statin alone and the bempedoic acid–statin combination may not reflect the pharmacology of the bempedoic acid–statin interaction but the multiplicative, rather than additive, mathematics of combining therapies with differing relative efficacy expressed in proportional terms. For example, if two lipid-lowering therapies both reduce LDL-C by 50% when administered as monotherapy, the effect of their combination is a 75% reduction, not 100% as would be derived by adding the percentages. Nevertheless, bempedoic acid provided a significant additional benefit compared with statins alone. Further, these data indicate that the addition of bempedoic acid to statins markedly increases the probability of achieving LDL-C goals.

One of the mechanisms implicated in muscle-related effects of statins is increased systemic exposure to statins, leading to increased uptake into skeletal muscle.^26^ Reducing the statin dose by 50% in the presence of bempedoic acid is predicted to reduce the systemic exposure of statin overall by about 25% because of the pharmacokinetic interaction with bempedoic acid, and, by extension, is also likely to lower the risk of myotoxicity. Although bempedoic acid acts on the same cholesterol biosynthesis pathway as statins, bempedoic acid is not activated in skeletal muscle and its addition to statin treatment is, therefore, unlikely to exacerbate statin-related muscle symptoms.^27^ Among patients in phase 3 studies receiving maximally tolerated statin therapy, myalgia was reported by 2.9% of patients receiving bempedoic acid vs. 3.1% of patients receiving placebo, and muscle weakness was reported by 0.4% of patients receiving either add-on bempedoic acid or placebo.^8^

The current study has some limitations. The model-based predictions from this study need to be validated further in clinical studies where the statin dose is reduced. The model was derived from non-uniform patient populations and may differ according to the type of dyslipidaemia diagnosed. Uncertainty added into estimating individual baseline pre-statin LDL-C assumes a population distribution of statin responses. However, the use of data from patients in clinical trials who have not achieved their goals in lowering LDL-C while taking a statin could have resulted in a selection bias towards those patients taking statins who responded poorly. Therefore, the estimated pre-statin baseline may have been different. Because of lack of appropriate studies to accurately characterize the interaction between statins and bempedoic acid, it was difficult to estimate the interaction coefficient. A sensitivity analysis was therefore used to fix an interaction coefficient in the model that best described LDL-C lowering by bempedoic acid in the presence/absence of each of the statins. If we could estimate the interaction coefficient and associated confidence interval, we could have made a definitive interpretation of the bempedoic acid–statin interaction. Also, other non-statin therapies were not considered in this model. Effects of covariates (gender, age, presence or absence of familial hypercholesterolaemia) were not evaluated in the present analysis. The data included in the model were from 12-week treatment, so that the combined model was unable to predict longer-term LDL-C lowering with bempedoic acid and reduced doses of statins. Furthermore, the data for bempedoic acid and statin combinations are almost entirely modelled from patients who received a bempedoic acid dose of 180 mg; therefore, there may be greater uncertainty in the predicted effect of bempedoic acid–statin combinations at other bempedoic acid doses.

## Conclusions

Dose–response models predicted meaningful reductions in LDL-C when bempedoic acid was combined with several statin dose intensities; therefore, the combination of bempedoic acid with lower dose statins provides therapeutic options for patients who cannot tolerate high-intensity statins or achieve LDL-C thresholds with maximally tolerated statin therapy alone.

## Supplementary Material

pvab064_Supplemental_File

## Data availability statement

To protect patient privacy, the data, analytical methods, and study materials will not be made available to other researchers for purposes of reproducing the results or replicating the procedures.
