# Supplementary material for: Pharmacodynamic effect of bempedoic acid and statin combinations: predictions from a dose–response model
Source: Eur Heart J Cardiovasc Pharmacother. 2021 Aug 27;8(6):578–86. doi: 10.1093/ehjcvp/pvab064 (PMC9440868; doi:10.1093/ehjcvp/pvab064)
Supplement: pvab064_Supplemental_File [file pvab064_supplemental_file.docx]

**SUPPLEMENTARY MATERIALS**

**Detailed Methods**

### Subjects

The 14 clinical studies included in the model enrolled healthy adults (1 study), adults with dyslipidemia/hyperlipidemia (11 studies), or adults with diabetes (2 studies). Separate modeling datasets were derived for each of the model conditions (ie, bempedoic acid monotherapy, bempedoic acid + atorvastatin, bempedoic acid + rosuvastatin, bempedoic acid + simvastatin, bempedoic acid + pravastatin) from the source clinical trial data. There were a total of 1105 patients included in the bempedoic acid monotherapy dataset with 2717, 1625, 1511, and 1304 patients in the combination datasets with atorvastatin, rosuvastatin, simvastatin, and pravastatin, respectively. Patients in the bempedoic acid monotherapy dataset were also included in the combination modeling datasets. There were 24 healthy subjects included in the dataset from a single study of bempedoic acid monotherapy, which included the high end of the dose range (eg, 140-260 mg). The remaining patients either had dyslipidemia or diabetes. The proportion of patients with heterozygous familial hypercholesterolemia ranged from 4.3% to 7.6% across the datasets. Clinical atherosclerotic cardiovascular disease status was not captured in the modeling dataset. The proportion of older adults (>65 years) ranged from 27.9% to 43.0% across the datasets and 44.6% to 45.5% were obese with a body mass index 30.0 kg/m^2^ or greater.

### Determination of pre-statin baseline

Baseline low-density lipoprotein cholesterol (LDL-C) concentrations for patients receiving bempedoic acid monotherapy and those receiving combination treatment with statins were not equivalent in the included studies. The patients enrolled into bempedoic acid and statin combination treatment arms were already receiving stable statin therapy at study entry; therefore, their baseline LDL-C concentrations reflected the concentration following stable statin therapy. Conversely, baseline LDL‑C concentrations in patients receiving bempedoic acid monotherapy represent the true baseline before any lipid-lowering therapy was administered. To pool these data for analysis, baseline LDL-C concentration measured at study entry had to be extrapolated to the “pre-statin” baseline for patients receiving combination therapy, which was accomplished by shifting the relative time scale by 5 weeks, so that the observed baseline in the clinical study was adjusted to week 5. Five weeks of elapsed time was deemed sufficient for maximal response to statin treatment to have occurred.^1^ Pre-statin baseline LDL-C concentrations were computed using statin monotherapy dose-response model parameters obtained from the literature.^2^ A parametric bootstrapping procedure was used to incorporate uncertainty in the individual model parameter estimates.

### Structural model

An indirect response-model structure was used to quantitatively describe the effect of bempedoic acid alone or in combination with individual statins on concentrations of low-density lipoprotein cholesterol (LDL-C) given that the relationship was described by individual time-course data. Model predictions of LDL-C concentration were determined by the rate of production (K_in_) and rate of elimination (K_out_), as presented below.


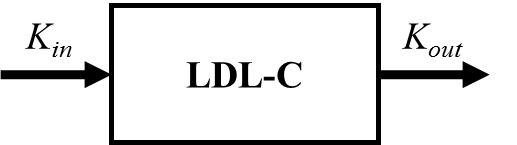


When no drug is present, the response (R) with time can be described by the following equation:

$$\frac{dR}{dt}=K_{in}-K_{out}\cdot R,$$

where K_in_ is a rate constant representing zero-order production of LDL-C, K_out_ is a first-order rate constant describing the degradation of LDL-C, and R is the LDL-C concentration.

The drug effect was described by following equations:

$$\frac{dR}{dt}=K_{in} \cdot\left( 1-E_{total} \right)-K_{out}\cdot R$$

and

$$E_{total}=E_{BA}+ E_{statin}+\left( \gamma\cdot E_{BA}\cdot E_{statin} \right),$$

Where E_BA_, E_statin_, and E _total_ represent fractional inhibition of LDL-C by bempedoic acid, the individual statin, and the combination of bempedoic acid and the individual statin, respectively. The interaction coefficient (γ) describes the interaction between bempedoic acid and the individual statin. This equation is analogous to the one used in the dose-response meta-analysis model developed for individual statins.^2^ A sigmoidal E_max_ (maximal drug effect) model was used to characterize the dose-response relationship for bempedoic acid and each individual statin separately:

$$E_{drug}=\frac{E_{max}\cdot{Dose}^{Hill}}{{{ED}_{50}}^{Hill}+{Dose}^{Hill}} ,$$

where E_drug_ is the drug effect (of bempedoic acid or individual statin), E_max_ is the maximal drug effect, Dose is the dose of the drug, ED_50_ is the dose of the drug needed to achieve 50% of the maximal drug effect, and Hill is the sigmoidicity coefficient describing the steepness of the dose‑response relationship.

### Random effects model

Inter-individual variability in model parameters was incorporated where applicable using a log‑normal, random-effects model of the form:

$$\theta_{i}{=\theta}_{TV}\cdot e^{\eta_{i}},$$

where *θ_i_* is the individual value of the model parameter (eg, baseline LDL-C) for *i^th^* participant, *θ_TV_* is the typical value model parameter in the population, and *η_i_* denotes the inter-individual random effect accounting for the *i^th^* individual’s deviation from the typical value. The distribution of *η_i_* is assumed to be normal with a mean zero and variance *ω^2^*. Thus, the approximate percent coefficient of variation is calculated as:

$$\%CV= \sqrt{\omega^{2}}\cdot100\%.$$

Residual or within-individual variability was modeled using an additive and proportional error model as described in the following equation:

$$Y_{ij}=R_{ij}\cdot\left( 1+\varepsilon_{1ij} \right)+\varepsilon_{2ij},$$

where *Y_ii_* denotes the observed LDL-C concentration for the *i^th^* individual at time *t_j_, R_ij_* denotes the corresponding individual predicted LDL-C concentration based on dose-response model, and 𝜀_𝑖𝑗_ denotes residual random effects, which is assumed to be normally distributed with mean zero and variance σ^2^.

### Combination model

Following identification of an appropriate bempedoic acid monotherapy dose-response model, combination models were developed individually for each of the four statins of interest: atorvastatin, rosuvastatin, simvastatin, and pravastatin. Model parameters describing the effect of bempedoic acid were fixed to the final estimated values from the monotherapy dose response model. Statin effect parameters were obtained from the previously published model^2^ and fixed to their mean values for each individual statin model while baseline LDL-C, the bempedoic acid–statin interaction coefficient, and random-effect parameters were initially estimated.

### Interaction coefficient sensitivity analysis

Examination of goodness-of-fit plots revealed some potential misfit when the bempedoic acid–statin interaction coefficient (γ) was estimated individually for each statin. Furthermore, it is believed that the value of this interaction coefficient describing the bempedoic acid–statin interaction should be consistent across individual statins owing to their common mechanism of action.^2^ To address these considerations, simulations were performed at various fixed-dose combinations of bempedoic acid (0 and 180 mg) with atorvastatin (0, 2.5, 5, 10, 15, 20, 40, 60, and 80 mg) while varying the value of the interaction coefficient. The interaction coefficient that best described the observed percent change from baseline in LDL-C at week 12 was used as a fixed parameter in subsequent analyses. Goodness-of-fit plots were used to visually evaluate the impact of a single fixed value for this interaction coefficient on model predictions for individual statins.

**References**

1. Kakara M, Nomura H, Fukae M, Gotanda K, Hirota T, Matsubayashi S, Shimomura H, Hirakawa M, Ieiri I. Population pharmacodynamic analysis of LDL-cholesterol lowering effects by statins and co-medications based on electronic medical records. *Br J Clin Pharmacol* 2014;**78**:824-835.

2 Mandema JW, Hermann D, Wang W, Sheiner T, Milad M, Bakker-Arkema R, Hartman D. Model-based development of gemcabene, a new lipid-altering agent. *AAPS* J 2005;**7**:E513-522.
